# Supplementary material for: Asymmetric and adaptive reward coding via normalized reinforcement learning
Source: PLoS Comput Biol. 2022 Jul 21;18(7):e1010350. doi: 10.1371/journal.pcbi.1010350 (PMC9345478; doi:10.1371/journal.pcbi.1010350)
Supplement: S1 Appendix — (DOCX) [file pcbi.1010350.s001.docx]

**S1 Appendix**

*Analytic derivation of NRL value function inflection point*

The NRL value function is parameterized by two parameters, an input exponentiation term *n* and a semisaturation term *σ*. The critical feature of the NRL algorithm that generates variable asymmetries in RPE coding is the presence of an inflection point between convex and concave value coding regimes. Here we show that an inflection point exists for any *n* > 1. The NRL value function is defined as:

$U(R)=\frac{R^{n}}{\sigma^{n}+R^{n}}$ (S1)

The first derivative of the value function taken with respect to *R*, obtained via the quotient rule and simplification, is:

$U^{'}\left( R \right)=\frac{no^{n}R^{n-1}}{{(\sigma^{n}+R^{n})}^{2}}$ (S2)

Similarly, the second derivative is:

$U^{''}(R)=\frac{n\sigma^{n}R^{n-2}(\left( n-1 \right)\sigma^{n}-\left( n+1 \right)R^{n})}{{(\sigma^{n}+R^{n})}^{3}}$ (S3)

An inflection point *R*^*^, defined here as the value of *R* when the second derivative transitions from positive (convex value function) to negative (concave value function), thus occurs when *U^’^*^’^ = 0. For 0 < *n* ≤ 1, there are no zeros of the second derivative equation since the numerator term $\left( n-1 \right)\sigma^{n}-(n+1)R^{n}$ is always negative and thus *U^’’^* is always negative and the value function is always concave. However, for *n* > 1, the value of the inflection point *R*^*^ is given by:

$R^{*}=\sqrt[n]{\frac{n-1}{n+1}}\sigma$ (S4)

The inflection point equation explains the relationship between RPE asymmetry and *σ*. Because the inflection point is a linear function of *σ*, increasing *σ* will tend to shift reward coding from concave to convex regimes and RPE asymmetries from negatively to positively biased. Note that at biologically plausible values of *n* (~2), the inflection point will occur at rewards lower than *σ*; at large *n*, the sigmoidal shape of the normalized value function approximates a step function and R* will approach *σ*.

*NRL agent behavior in the variable reward magnitude task*

To compare NRL model predictions to empirical dopaminergic neuron data, we quantified NRL responses in a variable reward magnitude task in which one of size potential rewards were randomly delivered. In this task, empirical dopamine responses exhibit a number of features consistent with distributional RL: (1) a diversity of biases in negative versus positive RPE coding, (2) a diversity of reversal points in the same reward environment, (3) a relationship between reversal point and RPE asymmetry, and (4) information allowing a decoding of the experienced reward distribution.

For the NRL model, we examined the behavior of a population of NRL agents with heterogeneous *σ* values (*N*=40); the number of agents was chosen to match the number of empirically recorded neurons in recently published work. Semisaturation values were chosen to evenly span a large range of values (0.5 to 48 A.U.), but altering the precise spacing and range of semisaturation values does not qualitatively change any results.

To address NRL behavior in the variable reward magnitude environment, we quantified the *reversal point* and the *RPE asymmetry* of each NRL agent; these two features are critical inputs to the expectile-based distribution decoding algorithm (see below). In our primary approach, we first derived the analytical steady state NRL response functions and then estimated the associated reversal points and RPE asymmetries. The response of an NRL agent at time *t* is given by:

$A_{t}=\frac{R_{t}^{2}}{\sigma^{2}+R_{t}^{2}}-V_{t}$ (S5)

representing the difference between the nonlinear transformation of received reward *R_t_* and the current value estimate *V_t_* (updated via Eqn. 3). At steady state, we assume that a given NRL RPE channel learns a value estimate that is the average of the transformed inputs (from the set *r* = {0.1, 0.3, 1.2, 2.5, 5, 10, or 20 A.U.}:

$\bar{V}=\frac{1}{7}\sum_{j=1}^{7} \frac{r_{j}^{2}}{\sigma^{2}+r_{j}^{2}}$ (S6)

with the corresponding steady state NRL response function (representing RPE) given by:

$$A^{*}=\frac{R^{2}}{\sigma^{2}+R^{2}}-\bar{V}$$

The reversal point *R*^*^ is the reward at which the RPE function, given by NRL agent activity, transitions from negative to positive values. This point is defined at steady state by setting *A** to 0 and solving for *R*:

$R^{*}=\sigma\sqrt{\frac{\bar{V}}{1-\bar{V}}}$ (S7)

Once the reversal point was identified, we quantified RPE asymmetry via piecewise linear regression for points below and above the reversal point. The slopes of these regressions were denoted *α^-^* and *α^+^,* and the RPE asymmetry defined as in previous work as:

$\tau=\frac{\alpha^{+}}{\alpha^{+}+\alpha^{-}}$ (S8)

As shown in Fig. 4, NRL reversal points and RPE asymmetries are related in a manner consistent with both theory and empirical data: different NRL agents show different reversal points, these reversal points are controlled by NRL parameterization (*σ*), and reversal points are correlated with asymmetries.

*Distribution decoding*

Structured diversity in RPE asymmetries have been proposed to support distributional RL, in which a learning system learns the full probability distribution of future rewards. We examined whether a small population of NRL agents (*n*=40) can learn distributional information in four different reward environments (rewards hereafter in A.U.): (1) symmetric, (2) right-skewed, (3) left-skewed, and (4) and multimodal. For the symmetric distribution, sample rewards were drawn from a Gaussian distribution (mean = 10, variance = 1). For the right-skewed distribution, sample rewards were drawn from a Pearson distribution (mean = 5, variance = 2, skewness = 1, kurtosis = 3); sample rewards for the left-skewed distribution were drawn from the same distribution and reflected across the value 10. In the first three environments, we generated 100 sample rewards; in the multimodal environment – constructed to replicate conditions under which empirical dopamine responses were recorded – samples were the set of rewards {0.1, 0.3, 1.2, 2.5, 5, 10, 20}. In each environment, these rewards served as sample points from the true distribution.

We implemented an expectile-based distribution decoding procedure that identifies (imputes) a reward density consistent with a given set of statistics learned from the true distributions. In each reward environment, a reward asymmetry *τ_n_* and reversal point *R_n_*^*^ was determined for each of the *N* = 40 NRL agents (as described above; *i* indexes individual agents). To perform distribution decoding, we interpreted the asymmetries as expectiles, where the *τ_n_*-th expectile had the value $R_{n}^{*}$. Decoding involved identifying a probability density that best mtached the statistics implied by the NRL agents, namely the set of expectiles. Specifically, we parameterized the density as a set of *M* = 100 reward samples *z_m_* and solved the minimization problem:

$\underset{z_{1},\ldots,z_{m}}{arg min} \mathcal{L}\left( z,R^{*},\tau\right)$, (S9)

$\mathcal{L=}\frac{1}{M}\sum_{m=1}^{M} \sum_{n=1}^{N} \left| \tau_{n}-\mathbb{I}_{z_{m}-R_{n}^{*}} \right|\left( z_{m}-R_{n}^{*} \right)^{2}$ (S10)

As in previous work, we first generated 20000 random samples for (*z*_1_,…, *z_m_*) uniform in the range [-5, 25] and initialized the minimization problem with the set of 100 sample points with the smallest loss. We solved this minimization problem using the MATLAB *fminunc* function, which performs unconstrained minimization, resulting in 100 reward samples that represent the decoded (or imputed) distribution. For purposes of display, the true and decoded distributions were smoothed via kernel density estimation.

*Equivalence of semistaturation and input weighting NRL parameterizations*

In the NRL model presented in the main text, parametric variability in RPE asymmetry is achieved via the semisaturation term *σ*. However, past work on the normalization models has proposed a role for the semisaturation term in adaptation. In such models, *σ* is history dependent and carries information about the average of recent inputs (i.e. rewards in the NRL framework). To preserve the *σ* term for capturing adaptation effects, an alternative but equivalent NRL formulation arises by implementing RPE asymmetry via a separate bias parameter *β*:

$U\left( R \right)=\frac{R^{n}}{{(\beta\sigma)}^{n}+R^{n}}$ (S11)

In this formulation, assuming a fixed *σ*, varying *β* changes the effective semisaturation term *βσ* in a manner analogous to the effects described in the main text. Mathematically, multiplying *σ* by *β* is equivalent to dividing the input rewards *R* by 1/*β*:

$U\left( R \right)=\frac{{(R/\beta)}^{n}}{\sigma^{n}+{{(R}/{\beta)}}^{n}}$ (S12)
