# Supplementary material for: Asymmetric and adaptive reward coding via normalized reinforcement learning
Source: PLoS Comput Biol. 2022 Jul 21;18(7):e1010350. doi: 10.1371/journal.pcbi.1010350 (PMC9345478; doi:10.1371/journal.pcbi.1010350)
Supplement: Attachment — Submitted filename: Louie - NRL PLOSCompBio response ro reviewers.docx [file pcbi.1010350.s002.docx]

We thank the Reviewers of the previous submitted manuscript for their thoughtful and helpful comments, and have incorporated their suggestions into our revision of the manuscript. We believe these changes have made the paper more readable and convincing. Below, we detail the changes and address each point raised by the Reviewers individually.

We note that in the previous version of the paper, the RL learning rate was referred to as both *α* and *η*, following previous usage in past papers. To improve consistency throughout the paper, we have adopted the same term for learning rates for all equations and figures (*η*), and reserve *α* for the slope of RPE coding (consistent with usage in the Dabney et al. paper).

All significant changes in the tracked version of the revised manuscript are highlighted in blue text.

**Reviewer #1:**

**Thanks for the opportunity to review the paper titled “Asymmetric and adaptive reward coding via normalized reinforcement learning”. The paper proposes a divisive normalization model of subjective value to explain important processes characterizing reinforcement learning. Simulations aim at demonstrating that the non-linear value function proposed by the model can explain (i) asymmetries between positive and negative learning rates and (ii) data about dopaminergic neurons. Overall, the paper is clearly written, and its contribution is timely and valuable. Below, please see some suggestions on how to improve the paper further:**

We thank the Reviewer for their careful reading of our manuscript and thoughtful suggestions for improvements. The major points raised - placing the contributions of the NRL model in the context of existing literature, clarification of the possible origins/meaning of model parameters, demonstrating the role of sigma in risk sensitivity, and providing more detail about the distributional simulations – are good ones, and we detail below our specific revisions in response to these points.

**Major points:**

**The author should clarify the specific contribution of the paper to the literature. It seems to me that the idea of proposing a reference-dependent sigmoid function (albeit in a different form) to describe subjective value is not novel (e.g., Woodford, 2012; Rigoli, 2019). The author should discuss this literature and clarify what the present paper adds to it. In my opinion, while previous literature adopting similar sigmoid models focuses on reference effects, the novel contribution of the paper is to explore the implications of a sigmoid value function in the context of RL; and show how this can explain (i) reported asymmetries in learning rate and (ii) recent data about dopamine function. Does the author agree on this interpretation? In any case, it would be helpful to clarify the precise contribution of the paper.**

We thank the Reviewer for both the interpretation of the paper’s contribution and the suggestion to discuss the NRL model’s place in the literature. The Reviewer is correct that reference-dependent sigmoid shaped functions for value coding are not new in the literature. As noted by the Reviewer, Woodford (2012) proposed an explanation for reference-dependent risky choice behavior, based on his existing theory that perception is intrinsically limited by processing constraints and behavior involves adaptation to the statistical theories of the environment, while Rigoli (2019) proposed a descriptive logistic model of reference-dependent contextual value coding. More generally speaking, other forms of context-dependence have been proposed, including reference point centering (Palminteri et al., 2015; Palminteri and Lebreton, 2021), range adaptation (Bavard et al. 2018, Bavard et al. 2021), and range frequency based (Hayes and Wedell, 2021) models.

We agree with the Reviewer that the application of a nonlinear, reference-dependent value function to RL, and the explanation of behavior and dopamine responses, are important contributions of the paper. Upon further consideration, we believe the NRL model proposed here contributes to this existing literature in four main ways. First, as noted by the Reviewer, it examines the implications of an S-shaped value function in a reinforcement learning framework. Second, the NRL model is based in neurobiological coding principles, in contrast to models intended to primarily characterize behavior. Specifically, the NRL model is derived from empirical findings on neural firing rates during decision-related reward coding (Louie et al. 2011, Eshel et al. 2015, Eshel et al. 2016, Yamada et al. 2018), takes its mathematical form from a canonical computation pervasive in sensory processing (Carandini & Heeger, 2012), and can be implemented in a biologically plausible way in simple neural circuits (Heeger & Zemlianova, 2020; Kouh & Poggio, 2008; Louie, LoFaro, Webb, & Glimcher, 2014). Third, a central and novel feature of the NRL model is parametric diversity in the degree of RPE asymmetry, an effect that explains variability in both behavior and neural responses. Finally, the combination of adaptation and distributional coding in the NRL suggests the possibility of rich contextual reward learning, offering a single biological mechanism for diverse phenomena currently explained by different reference point centering (Palminteri, Khamassi, Joffily, & Coricelli, 2015), range adaptation (Bavard, Lebreton, Khamassi, Coricelli, & Palminteri, 2018), and range frequency based (Hayes & Wedell, 2021) models.

To address this, we have included the following text in the Discussion that touches upon existing work on reference-dependent sigmoidal value functions and outlines the specific contributions of the current paper:

“Finally, context-dependent in valuation has been a recent area of interest in psychology, economics, and neuroscience, and it is important to consider how the NRL model relates to existing models of context-dependent valuation and choice. In its most detailed implementation (Eqn. 5), NRL provides a parametric control over both contextual adaptation (via *σ*) and RPE asymmetry (via *w*), implementing a reference-dependent S-shaped value function similar to several previously proposed models. Most broadly, prospect theory and related models propose an asymmetric value function convex in the region of losses and concave in the region of gains, with gains and losses determined relative to a reference point (Kahneman & Tversky, 1979; Koszegi & Rabin, 2006). More recently, similar value functions arise in models derived from efficient coding principles in response to processing constraints (Woodford, 2012) or designed to capture adaptation effects in choice behavior (Francesco Rigoli, 2019). While the NRL value function also exhibits an S-shaped reference-dependence, it extends previous work in a number of ways. First, it applies nonlinear valuation to reinforcement learning, where linear reward functions are widely and conventionally employed; in contrast to prior behavioral theories, the NRL framework makes direct and testable predictions about both behavior and dopaminergic neural activity. Second, the normalized value function has a clear biological grounding: it defined from neurophysiological reward responses (Louie et al., 2011; Yamada, Louie, Tymula, & Glimcher, 2018), based on a canonical neural computation (Carandini & Heeger, 2012), and implementable in simple neural circuits (David J Heeger & Zemlianova, 2020; Kouh & Poggio, 2008; Louie, LoFaro, Webb, & Glimcher, 2014). Third, the NRL algorithm incorporates a novel parametric control of the degree and direction of asymmetric responding to negative and positive RPEs; diversity in NRL RPE asymmetry provides a unitary explanation for diversity in both behavior and neural responses. Finally, the combination of adaptation and distributional coding suggests the possibility of rich contextual reward learning, offering a single biological mechanism for diverse phenomena currently explained by different reference point centering (Palminteri, Khamassi, Joffily, & Coricelli, 2015), range adaptation (Bavard, Lebreton, Khamassi, Coricelli, & Palminteri, 2018), and range frequency based (Hayes & Wedell, 2021) models.”

**Although the paper hints to reference effects in shaping the value function (also consistent with the literature on normalization mentioned in the introduction), the nature of these effects is not explained. Specifically, the paper is silent about where the parameters sigma and n come from. Do they depend on the property of the distribution of rewards (e.g., sigma being an average and n being an index of variability)? I think that an in-depth analysis of this aspect is not necessary here, as the main points of the paper emerge clearly enough as it is. Yet, a short clarification of this important aspect of the model would be beneficial. Relatedly, given that the focus is on RL, the discussion might briefly speculate on how the parameters are themselves acquired via learning.**

The Reviewer raises an important point about the source of the parameters, in particular for the full version of the NRL model provided in Equation 4. This specification involves three parameters: the exponent *n*, the input weighting factor *w*, and the semisaturation term *σ*. The exponent is the most rigid: the general findings reported here (convex-concave transition, asymmetric responses for positive and negative prediction errors) occur for any exponent >1, and in most normalization models the parameter is generally assumed to be 2. This value matches fits of the normalization equation to neurophysiological data which produce exponents between 2 and 3 (Carandini and Heeger, 2012), and is consistent with the original interpretation of a quadratic exponentiation of input as an approximation to threshold linear spiking in individual neurons (Heeger, 1992). In this paper, we take the NRL exponent *n* as fixed at 2.

In contrast, the input weighting and semisaturation parameters have a freer parameterization and more interesting conceptual implications. In the full specification, the input weighting term *w* shifts the nonlinear reward function relative to *σ* and serves to govern parametric control of RPE asymmetry. As the Reviewer notes, the semisaturation term can now implement adaptation by incorporating information about past rewards. Thus, to answer the question directly, in the full parameterization the exponent *n* is considered fixed, the weight *w* term is heterogeneous but stable (across neurons and people), and the semisaturation term *σ* is history-dependent.

To address the Reviewer’s concerns for clarity about parameter contributions and possible learning, we have added additional text, including text to address the nature of the exponent in the Results (*Normalized reinforcement learning model*):

“The exponent *n* in normalization models governs amplification of inputs, and is generally considered a fixed property of the model (Carandini & Heeger, 2012; Heeger, 1992).”

To address the semisaturation parameter in regards to adaptation and reference-dependence, we have expanded the relevant text in the Results (*Alternative parameterizations and biological plausibility*):

“Importantly, this alternative formulation also preserves the ability of the NRL model to capture adaptation, analogous to a reference point in history-dependent models of context-dependent decision-making. In sensory neuroscience, adaptation in neural responses is typically implemented in normalization approaches via a history-dependent *σ* term that accounts for recent past stimuli. For example, normalization models with a dynamic *σ* term that averages past contrast levels explains adapting responses in visual cortical neurons (17) and has been shown to improve efficient coding via redundancy reduction (37). Furthermore, the adaptation of human valuation behavior is captured by a normalization mechanism with an equivalent *σ* averaging past rewards (38). Thus, by separating how the NRL algorithm models RPE asymmetry and reward history, this alternative parameterization can be used to examine neural and behavioral adaptation effects during reward learning.”

Finally, we include new text in the discussion to summarize NRL parameters and briefly discuss possible mechanisms for learning a dynamic semisaturation term for reference-dependent valuation:

“In its fullest form (Eqn. 5), the NRL algorithm is governed by three parameters with specific neurobiological interpretations and different functional implications. The exponent *n* governs input amplification, and is generally viewed as a fixed property of normalization computations. The input weighting term *w* implements parametric diversity in RPE asymmetries, which underlies the behavioral and neural diversity generated by the NRL model. Though a novel feature of the model, input weighting diversity may be simply realized as heterogeneity in synaptic strength or number of afferents. Finally, as in standard models of divisive normalization, a dynamic semisaturation term *σ* can implement reference-dependent valuation by adjusting the normalized value response to recent rewards. The precise mechanism by which *σ* adapts is not known, but possibilities include changes in pooled inputs contributing to normalization (17), long timescale nonlinear dynamics in divisive gain control (39, 40), and RL-like computations learning average reward. Regardless of mechanism, an adaptive *σ* allows the NRL model to flexibly encode value in changing reward environments, adjusting reward coding relative to a reference point implemented as the *σ* parameter.”

**As argued above, the simulation described in fig. 3 appears crucial to me, as it reflects one of the main contributions of the paper (explaining asymmetries in learning rates as arising from non-linear value functions). However, I am not sure how robust the result that sigma inversely correlates with risk aversion is. Does this also depend on the range of rewards simulated? I suggest to vary the reward distribution (keeping the parameters fixed) in different simulations and show what happens. In general, a few other simulations on this point (e.g., based on empirical studies conducted by Palminteri’s lab or Frank’s lab) would make the argument more sconvincing. Relatedly, what is the role of the parameter n in risk sensitivity?**

The Reviewer raises an important point about the relationship between reward magnitudes and the semisaturation term (in the simple parameterization of the NRL model; equivalently, the following statements apply to *w* in the full NRL parameterization [Eqn. 5]). First, we wish to emphasize that the important takeaway from the results in Figure 3 is not the specific relationship between the NRL semisaturation term and behavioral risk aversion, but the general finding that variability in risk aversion and implied learning rates can be generated by changes in the nonlinear NRL value function. While we demonstrate a strong negative correlation between the semisaturation term and risk aversion (Fig. 3C), this figure is largely intended to highlight for the reader the fact that variability in risk aversion in these simulations arise from NRL parametric diversity.

However, we acknowledge that understanding the relationship between risk sensitivity and the NRL model is an interesting and relevant point. The specific quantitative relationship between risk sensitivity and NRL parameters will depend on the relative magnitudes of outcomes and the semisaturation term. For example, if reward magnitudes are very large (i.e. both the certain and the risky outcomes are >> the range of *σ* being examined), then all agents will exhibit risk aversive behavior; alternatively, if reward magnitudes are very small, all agents will demonstrate risk-seeking behavior. Thus, for a given NRL agent (or individual), behavior will exhibit more risk aversive behavior for larger rewards, a point briefly touched upon in the Discussion. In terms of replicating the analysis in Figure 3, this magnitude dependence means that a full range of risk preferences (risk seeking and risk aversive) generally arises only when rewards and the semisaturation term of comparable magnitudes. At extremes of rewards relative to the semisaturation terms tested), behavior will be constrained to be all similarly risk seeking or risk averse, reducing any variability in behavior (and strong relationship to *σ*). This fact suggests a useful benefit of the full NRL parameterization (Eqn. 5), which can maintain a stable diversity of RPE asymmetry (via the *w* parameters) while adapting to reward magnitudes (via an adaptive *σ* term).

While we believe that the specific relationship between *σ* and risk aversion is not crucial in establishing that the NRL model can generate variable risk preferences, we nevertheless show below that the general relationship between *σ* and risk sensitivity is robust to specific reward magnitudes. As suggested by the Reviewer, we examined the relationship between the NRL *σ* term and behavioral risk aversion for different certain and risky outcomes, structured as in the task. Specifically, analogous to the simulations presented in Figure 3C, we generated a family of agents with semisaturation terms sampled from [10, 80] and quantified risk preference in the Niv et al. task for different reward magnitudes (a certain outcome of $X and a risky lottery with a 50% chance of $2X and a 50% chance of $0). Note that the same NRL agents (defined by a set of *σ* terms, *n*=2, and *σ*-dependent choice noise) were examined across different reward magnitudes; given the stochastic nature of behavior in these simulations, this process was repeated 20 times. Figure R1 shows that the inverse relationship between the NRL semisaturation term and behavioral risk aversion (evident in Figure 3D and quantified as a significant negative correlation in the main text) persists across different reward magnitudes. Note that the strength of the relationship decreases at low and high relative reward magnitudes for two reasons. First, as noted above, at extreme reward magnitudes (relative to the semisaturation terms tested) risk preferences will become more homogenous (either all risk seeking, or all risk averse); this compression reduces the relationship between NRL variability and variability in risk behavior. Second, without an explicit assumption about how noise in the decision process scales with reward magnitude, extremes of reward sizes will affect the choice process and the relationship between NRL *σ* and risk aversion at both small and large rewards. For small rewards, the difference between options becomes indistinguishable and all choice behavior approaches randomness; for large rewards, the difference between options is large and NRL agents exclusively pick the more valuable outcome. Both these effects reduce the ability to detect a relationship between NRL models and risk aversion.

**Figure R1. Effect of reward magnitude on relationship between NRL semisaturation term and risk preference.** A range of NRL agents (*σ* sampled from [10, 80]) were tested in a choice between a certain ($X) and a risky (50% $2X, 50% $0) outcome for different X values; simulations were conducted in the same manner as for the data shown in Figure 3 (learning rate η = 0.1, 1000 trials). Data plot the correlation between *σ* and behavioral risk aversion as a function of reward magnitude scaling (relative to X=20). Black circles show a set of simulations (a set of identical NRL agents simulated across different reward scalings); red points denote average correlations across 20 sets of simulations.

To briefly address these points in the revision, we have added the following text to the Results:

“Rather than the specific relationship between NRL parameterization and risk behavior, we emphasize that these results highlight the general finding that variability in risk aversion and implied learning rates can be generated by changes in the nonlinear NRL value function.”

Regarding additional simulations of other results, we agree with the Reviewer that simulating the NRL model in other tasks is a useful and important approach, and that a number of labs have published interesting and relevant findings related to RL models of risk preferences. For example, the Palminteri group has examined how asymmetric learning in RL models can fit human behavior in paradigms that include gains and losses (Palminteri et al., 2015), confidence judgments (Lebreton et al., 2019), adaptation (Bavard et al., 2018) and neuroimaging (Lefebvre et al., 2017). Similarly, the Frank group has examined asymmetric RL in diverse applications including schizophrenia (Gold et al., 2012; Dowd et al., 2016), genetic mechanisms (Frank et l. 2007), and Parkinson’s disease (Frank et al., 2006), and Pavlovian vs. instrumental learning (Swart et al., 2017). These findings represent a wealth of data that we see as next steps in developing the NRL model; however, at this time, we feel that exploring these extensions lie beyond the basic findings we wish to emphasize here: that parametric diversity in the NRL model is sufficient to explain variable risk preferences, and assumptions about linear reward functions can confound interpretations about variable gain/loss learning rates. If the Reviewer has particular findings that they believe are crucial to simulate, however, we would be happy to include them in a revision.

Regarding the role of the exponent *n* in risk sensitivity, it essentially plays a permissive role in generating the full range of variable risk sensitivity. That is, NRL value functions with *n*>1 will exhibit an inflection point and a convex-concave transition and exhibit qualitatively similar results to the simulations we show here. In contrast, NRL value functions with *n*≤1 will exhibit purely concave value functions; while such agents will still demonstrate variability in risk preferences, their behavior will be generally all risk averse. However, we wish to emphasize here that, as is standard in divisive normalization interpretations, we view the exponent as fixed term reflecting input amplification rather than a free parameter to fit to data; in fact, studies that have fit the normalization model to electrophysiological data reliably report the exponent to be between 2 and 3 (Carandini and Heeger, 2012).

**Regarding the paragraph starting with “Recent work shows that empirical dopamine responses exhibit...”, I found the description of the simulation unclear. Please provide all the relevant information about this simulation in this paragraph.**

We apologize that the details were insufficient in the original manuscript, and have revised the manuscript to provide details of the simulation approach. Briefly, we simulated different NRL agents in an environment matching the electrophysiological experiment that generated the dopaminergic data in the Dabney et al. paper (original data reported in Eshel et al., 2015). We have revised and expanded the description in the relevant paragraph with the following text:

“Recent work shows that empirical dopamine responses exhibit this hypothesized variability in reversal points, driven by asymmetric scaling of negative and positive RPE responses (Dabney et al., 2020). Given that such asymmetric RPE responses are intrinsic to NRL, we examined the relationship between different NRL agents with varying *σ* parameters and their reversal points in a stochastic outcome environment replicating the previously reported experiment (Eshel et al., 2015; Eshel et al., 2016). Specifically, we simulated NRL responses to single rewards drawn from a distribution of seven different reward magnitudes (0.1, 0.3, 1.2, 2.5, 5, 10, or 20 A.U.); each possible reward outcome was drawn with an equal probability, and we examined steady state NRL RPE responses across outcomes. We find that NRL agents simulated in such a stochastic reward environment exhibit a diversity of reversal points (examples, Fig. 4A) directly related to the individual NRL channel *σ* parameter (Fig. 4B).”

**Minor points:**

**Fig 1 b: please report the values of sigma and n adopted in the simulation.**

We have now added that information to the Figure 1 legend:

“In contrast to RL, NRL algorithms learn values that are a nonlinear function of external rewards (example NRL simulation parameters:$\sigma$= 50, *n* = 2).”

**Line 100: I think it should be “asymmetric prediction errors”**

This has been corrected.

**“Intuitively, these apparent learning rates arise because fitting with the standard RL model (Eqn. 4) approximates bipartite linear regression on the nonlinear value function”. This sentence is not clear to me.**

We agree that the previous wording lacked clarity, and that this is an important point to convey. When fitting data generated by a system with a single learning rate and nonlinear reward function, assuming two different learning rates (for gains and losses) and a linear reward function will (incorrectly) result in apparently different gain/loss learning rates. The key is that the asymmetry lies in the reward function itself, not the learning rates. We have now tried to convey this concept in revised text:

“Intuitively, these apparent learning rates arise because fitting with the standard RL model (Eqn. 4) and two valence-dependent learning rates approximates bipartite linear regression on the nonlinear value function. In other words, the different curvature of the NRL value function for negative and positive RPEs (as seen in examples in Fig. 3A) will be captured (incorrectly) as differential linear modulation by negative and positive learning rates.“

**Regarding the paragraph “Moreover, the intrinsic RPE asymmetry of NRL model..” the author nicely draws a parallel between the simulation’s results and the main four findings reported by Dabney at al (2020) outlined previously. It would help to make the parallel more explicit.**

We agree this is an important point, and have revised the text at the end of the paragraph to summarize and explicitly name the parallels between dopamine neurons and NRL agents:

“Thus, NRL agents replicate the key empirical features seen in dopamine neurons: a diversity of reversal points, variable RPE asymmetries, a strong reversal point-asymmetry relationship, and an encoding of the statistical distribution of experienced rewards. Together, these findings suggest that the NRL algorithm provides a robust computational mechanism for distributional RL.“

**Reviewer #2:**

**In the present study, the author describes behavioral and computational implications of using non-linear reinforcement learning (RL) models. The author characterized a RL model implementing a non-linear value function of reward via a divisive normalization computation. The author shows that this non-linear specification can produce asymmetrical prediction errors coding and then explain both behavioral phenomena such as attitudes toward risk and computational mechanisms underlying distributional learning in the brain. The paper is well written, clear, and concise, and the question raised by the author is of prime interest. The link made between non-linear value functions and updating asymmetry in linear models with multiple learning rates is particularly interesting and promising, and in making that link and describing its computational implication, the study fulfils its goal perfectly. The paper is very informative, and I enjoyed reading it. On a side note, it is not clear how the model, in its current version, will be useful to analyze behavior in future studies. The later can produce relevant effects observed in humans but the lack of clear view on what could represent (cognitively) or determine the semisaturation parameter is problematic. It is not obvious either whether the model can fit reliably human behavior and make actual predictions about the later.**

The Reviewer raises several important points and we thank them for their careful reading of the manuscript and thoughtful suggestions, especially about the functional and cognitive role of the semisaturation parameter and the potential to adequately capture human behavior. These are important conceptual and practical issues that we address in detail below.

**Major comments**

**#1 Alternative non-linear models**

**The non-linear valuation in the model can produce asymmetric prediction errors coding, but so would do other nonlinear, convex or concave, utility functions. For instance, another function that is convex then concave (like the present function for n≥2) is the prospect theory valuation function, which is convex below the reference point and concave above. Moving the reference point would then produce similar effects as moving the semisaturation parameter in the present model. Beyond its explanatory power at the neural level, could author explain the advantages of using the present model over other non-linear ones?**

The Reviewer is correct that previous work has proposed S-shaped utility functions. As mentioned in the review, prospect theory proposes a reference-dependent value function with a convex-concave transition at the reference point. As raised by the other Reviewer, more recent models (Woodford, 2012; Rigoli, 2019) propose similar (reference-dependent) S-shaped value functions.

In our view, there are two primary advantages conferred by the normalization model: biological grounding and parametric variability. In terms of biological grounding, the conceptual starting point of the NRL model is that normalized value coding is consistently found in neural circuits, leading to the hypothesis that a reinforcement algorithm employing this particular nonlinear computation should capture aspects of behavior and neurophysiology. In addition to its basis in biological reward coding, the NRL algorithm implements the canonical divisive normalization computation, which is widely found across numerous brain regions and which exhibits a number of useful computational properties (e.g. efficient coding, probabilistic representation, and winner-take-all competition). This biological grounding extends to potential implementation in neural circuits: a number of biophysical mechanisms have been proposed to implement the normalization computation, suggesting that the S-shaped NRL value function can arise via simple neural circuit architecture. Furthermore, unlike behavioral models like prospect theory - which raises the importance of a reference point without specifying how it might be instantiated at a neural level - the NRL model suggests simple biological mechanisms for implementing an adaptive reference point. Drawing from past work in sensory normalization, for example, adaptation to recent reward history in the full model (Eqn. 5) can be achieved via a semisaturation term equivalent to average recent input (reward) and implemented at the neural level via population background inputs.

Perhaps the most important aspect of the NRL nonlinear function is that it provides a specific mechanism of parametric diversity in RPE asymmetry (implemented in the full model by the input weighting term *w*). This simple addition provides a parametric way to control the degree and direction of RPE asymmetry, in a manner that explains both across-individual variability in behavior and across-neuron variability in reward learning; such parametric variability in RPE asymmetry is to our knowledge novel and not a component of any previous models or value functions. Furthermore, implementing RPE diversity via input weighting suggests immediately plausible biological mechanisms for such diversity, via for example heterogeneity in synaptic weights or afferent connectivity.

To emphasize the advantages of the NRL model, we have outlined these advantages in a new paragraph in the Discussion:

“Finally, context-dependent in valuation has been a recent area of interest in psychology, economics, and neuroscience, and it is important to consider how the NRL model relates to existing models of context-dependent valuation and choice. In its most detailed implementation (Eqn. 5), NRL provides a parametric control over both contextual adaptation (via *σ*) and RPE asymmetry (via *w*), implementing a reference-dependent S-shaped value function similar to several previously proposed models. Most broadly, prospect theory and related models propose an asymmetric value function convex in the region of losses and concave in the region of gains, with gains and losses determined relative to a reference point (Kahneman & Tversky, 1979; Koszegi & Rabin, 2006). More recently, similar value functions arise in models derived from efficient coding principles in response to processing constraints (Woodford, 2012) or designed to capture adaptation effects in choice behavior (Francesco Rigoli, 2019). While the NRL value function also exhibits an S-shaped reference-dependence, it extends previous work in a number of ways. First, it applies nonlinear valuation to reinforcement learning, where linear reward functions are widely and conventionally employed; in contrast to prior behavioral theories, the NRL framework makes direct and testable predictions about both behavior and dopaminergic neural activity. Second, the normalized value function has a clear biological grounding: it defined from neurophysiological reward responses (Louie et al., 2011; Yamada, Louie, Tymula, & Glimcher, 2018), based on a canonical neural computation (Carandini & Heeger, 2012), and implementable in simple neural circuits (David J Heeger & Zemlianova, 2020; Kouh & Poggio, 2008; Louie, LoFaro, Webb, & Glimcher, 2014). Third, the NRL algorithm incorporates a novel parametric control of the degree and direction of asymmetric responding to negative and positive RPEs; diversity in NRL RPE asymmetry provides a unitary explanation for diversity in both behavior and neural responses. Finally, the combination of adaptation and distributional coding suggests the possibility of rich contextual reward learning, offering a single biological mechanism for diverse phenomena currently explained by different reference point centering (Palminteri, Khamassi, Joffily, & Coricelli, 2015), range adaptation (Bavard, Lebreton, Khamassi, Coricelli, & Palminteri, 2018), and range frequency based (Hayes & Wedell, 2021) models.”

**I understand why the divisive normalization is used here but is it, in the present version, a good behavioral model? Regarding the prospect theory function, moving arbitrarily the reference point to account for various behaviors, without clear assumption about how it is set, would not make much sense. And the same could be said with moving the semisaturation parameter in the present model, which has furthermore a less clear definition. Could the author comment on this point?**

We agree that using parameters in the NRL model (or any model, for that matter) in an unconstrained manner would allow it to fit more datasets, without offering insight about underlying, generative processes. For clarity, in the full version of the model (Eqn. 5), the semisaturation term is assumed to be an adaptation term (e.g. reward history) and parametric diversity is implemented via the input weighting term *w*. If the Reviewer is referring to this model version, them the semisaturation term is – as in prospect theory - a reference point, for example an average of recent reward learned via an RL updating mechanism. We have added new text to clarify this interpretation in the Results (*Alternative parameterizations and biological plausibility*):

“Importantly, this alternative formulation also preserves the ability of the NRL model to capture adaptation, analogous to a reference point in history-dependent models of context-dependent decision-making. In sensory neuroscience, adaptation in neural responses is typically implemented in normalization approaches via a history-dependent *σ* term that accounts for recent past stimuli. For example, normalization models with a dynamic *σ* term that averages past contrast levels explains adapting responses in visual cortical neurons (17) and has been shown to improve efficient coding via redundancy reduction (37). Furthermore, the adaptation of human valuation behavior is captured by a normalization mechanism with an equivalent *σ* averaging past rewards (38). Thus, by separating how the NRL algorithm models RPE asymmetry and reward history, this alternative parameterization can be used to examine neural and behavioral adaptation effects during reward learning.”

If the Reviewer, however, is referring in general to the parameter that controls parametric NRL diversity (*σ* in the minimal model [Eqn. 3], *w* in the full model [Eqn. 5]), they raise an important point about interpretability and consistency. The most natural interpretation is in the full model, where the semisaturation term is a function of past rewards (e.g. average), and the weighting term *w* that implements diversity has simple potential biological interpretations such as synaptic weighting or density of inputs. We have included new text in the Discussion that briefly discusses this issue:

“The input weighting term *w* implements parametric diversity in RPE asymmetries, which underlies the behavioral and neural diversity generated by the NRL model. Though a novel feature of the model, input weighting diversity may be simply realized as heterogeneity in synaptic strength or number of afferents.”

**#2 Interpretation of the semisaturation parameter**

**Related to point #1, I find it hard to grasp the meaning of the semisaturation parameter, at the behavioral level. In the simulations presented in the paper, it seems that its only goal is to place the concave or convex portion of the valuation curve adequately on the range of objective values in order to generate a positive or a negative asymmetry in prediction error coding respectively. In the same way and as an example, does it make sense to define the semisaturation parameter as being twice as large as the upper bond of the range of objective value in the considered environment (e.g., the choice task described here)?**

The Reviewer is correct that the semisaturation term – in the initial, pared down version of the NRL model used to examine choice behavior (Figure 3) – is important because it shifts the point of inflection in the NRL value function (which is not identical to the semisaturation value but related to it; see analytical derivation in the SI). This allows the NRL model to capture either risk seeking or risk averse behavior for the same objective rewards, driven by the emphasis on negative or positive RPEs as intuited by the Reviewer. In our approach, this allows the NRL model to capture different kinds of risk preferences across individuals in the identical environment. However, in our view there is no particular normative answer for where the semisaturation term should be set or defined in a given environment, at least not without defining an objective function for which the behavior is meant to optimize.

However, in the final, extended formulation (Eqn. 5), there is a more intuitive interpretation of the semisaturation parameter and where it should/might be defined. In this version, parametric NRL variability is shifted to the input weighting term *w* and the semisaturation term is free to model the reward environment (as in standard models of normalization). In this case, a common interpretation is that the semisaturation term reflects past stimulus/reward history, a variable that can be learned by the system in multiple ways. We have included new text in the Results that briefly touches on this point:

“Importantly, this alternative formulation also preserves the ability of the NRL model to capture adaptation, analogous to a reference point in history-dependent models of context-dependent decision-making. In sensory neuroscience, adaptation in neural responses is typically implemented in normalization approaches via a history-dependent *σ* term that accounts for recent past stimuli. For example, normalization models with a dynamic *σ* term that averages past contrast levels explains adapting responses in visual cortical neurons (17) and has been shown to improve efficient coding via redundancy reduction (37). Furthermore, the adaptation of human valuation behavior is captured by a normalization mechanism with an equivalent *σ* averaging past rewards (38).”

We have also included new text in the Discussion that briefly touches on this point:

“Finally, as in standard models of divisive normalization, a dynamic semisaturation term *σ* can implement reference-dependent valuation by adjusting the normalized value response to recent rewards. The precise mechanism by which *σ* adapts is not known, but possibilities include changes in pooled inputs contributing to normalization (17), long timescale nonlinear dynamics in divisive gain control (39, 40), and RL-like computations learning average reward. Regardless of mechanism, an adaptive *σ* allows the NRL model to flexibly encode value in changing reward environments, adjusting reward coding relative to a reference point implemented as the *σ* parameter.“

**3# On the use of the model in behavioral studies**

**It is not clear from the paper how well the model could fit behavioral data and how well the parameters could be recovered from simulated data. For instance, in the behavioral part of the paper, the model is used to generate data in a choice task, could the simulated data be reasonably well fitted by the generative model and the parameters of the latter well recovered?**

We agree with the Reviewer that testing model parameter recovery is a necessary part of determining whether the NRL is a useful computational model of behavior. Because our primary goal was to quantify the implications of using an empirically-derived, nonlinear value function in an RL framework, rather than verifying the applicability if the NRL in modeling behavior, we did not include these analyses in the original version of the paper. To address this issue, we show here simulation results below examining parameter recovery in the Niv et al. task described in Figure 3. As in the main results, we generated NRL agent behavior in the certain/risky choice task (1000 trials); these simulations were repeated 500 times, with the generative semisaturation and learning rate parameters drawn from uniform distributions (*σ*: [10, 80]; $\eta$: [0.05, 0.15]). As shown in Figure R2 below, simulated and fit parameters are strongly correlated with no bias (*σ*: *r* = 0.969; $\eta$: *r* = 0.923).

The suitability if the current NRL model for examining behavior is an important question, one that we plan on exploring more fully in future work; we thank the Reviewer for raising this issue. We note here that testing this suitability will require examining NRL and behavior in many scenarios beyond the simple two-option, risky/certain gamble presented in the Niv et al. work and this manuscript. The ability of the NRL to model behavior will likely depend on multiple factors, such as the reward distributions employed in a task and the number of trials and amount of data to be fit. For example, the correlations reported above increase (*σ*: *r* = 0.994; $\eta$: *r* = 0.988) if the models are simulated/fit with a larger number of trials (10,000).

**Figure R2. NRL parameter recovery.** 500 NRL agents were simulated in the Niv et al. choice task, replicating the conditions described in the main text and shown in Figure 3. The semisaturation term and learning rate were drawn from uniform distributions (*σ*: [10, 80]; $\eta$: [0.05, 0.15]); the choice rule softmax temperature was determined as a function of *σ* as in the primary simulations. Data plot fit versus simulated parameters for the semisaturation term (left) and the learning rate (right). Both parameters show a strong correlation between fit and simulated parameter values (*σ*: *r* = 0.969; $\eta$: *r* = 0.923).

**Minor comments**

**#1 Figure 1b - parameters It would be useful for readers to make explicit parameters that have been used in figure 1b.**

This has been corrected in the Figure 1 legend:

“In contrast to RL, NRL algorithms learn values that are a nonlinear function of external rewards (example NRL simulation parameters:$\sigma$= 50, *n* = 2).”

**#2 Figure 3a parameters**

**Similarly, it would be useful for readers to know which parameters have been used to generate the risk-seeking and risk- averse agents from figure 3a. We know the range of values that has been used for all agents but not the values of these two particular cases.**

We agree this is important information, and have now added detail to the Results text:

“Given the relationship between *σ* and RPE asymmetry, the NRL model can generate either risk averse or risk seeking behavior (Fig. 3A, blue lines; example risk averse σ = 20, example risk seeking σ = 60).”

We have also clarified the Figure 3 legend:

“Example risk averse σ = 20 (top); example risk seeking σ = 60 (bottom).”
